# Current Balloon Devices for Ablation of Atrial Fibrillation

**DOI:** 10.31083/j.rcm2501034

**Published:** 2024-01-22

**Authors:** Shiro Nakahara, Yuichi Hori, Reiko Fukuda, Hirotsugu Sato, Hideyuki Aoki, Yuki Kondo, Yuta Kimura, Yuji Itabashi, Tetsuya Ishikawa, Sayuki Kobayashi, Isao Taguchi

**Affiliations:** ^1^Department of Cardiology, Dokkyo Medical University Saitama Medical Center, 343-8555 Koshigaya, Japan

**Keywords:** atrial fibrillation, cryoballoon, hot balloon, laser balloon

## Abstract

Balloon-based catheter ablation is a valuable option for the treatment of atrial fibrillation (AF) because contiguous lesions can be created to achieve pulmonary vein isolation (PVI), and the method is less dependent than traditional ablation methods on the operator’s skill and experience. Cryoballoon ablation is used universally worldwide, with its efficacy and safety being comparable to the efficacy and safety of standard radiofrequency ablation, and the procedure can be completed in a relatively short time. Hot balloon ablation was developed in Japan. The balloon maintains its compliance even during the energy delivery, and a large areal ablation lesion is created. Furthermore, the hot balloon system is the only system for which oesophageal cooling is a standard feature. Laser balloon ablation, which is performed under direct endoscopic vision, has proven to be effective and safe for achieving a PVI. The laser balloon system provides an improved field of view and automated circumferential ablation for a rapid and effective PVI. The authors have reviewed the currently available balloon systems as used for AF ablation, i.e., PVI, and have provided detailed insight and perspectives on the currently available cryoballoon and hot balloon technologies, plus laser balloon technology.

## 1. Introduction

Pulmonary vein isolation (PVI) by means of catheter ablation is the mainstay interventional treatment for both paroxysmal and persistent atrial fibrillation (AF) [1]. Although additional targets have been suggested, the standard initial approach is complete electrical isolation of all four pulmonary veins (PVs) [2]. Advances in radiofrequency (RF) catheter ablation techniques plus the addition of irrigation systems and the use of contact-force sensing and ablation indices to better determine the lesion size have improved the treatment outcomes [3, 4]. However, the catheter-based point-by-point approach requires considerable time and relies on the operator’s skill. Thus, the achievement of a reproducible and durable PVI remains challenging [3, 5]. Ineffective lesions, i.e., lesions with conduction gaps between the PV and left atrium (LA), can lead to recurrent AF or atrial tachycardia [6]. Balloon-based ablation techniques offer a “one-shot” solution and “shorter operator learning curve” that is less dependent on operator dexterity and creates a contiguous lesion for an effective PVI [7]. Furthermore, it is widely recognized that the characteristic softness of the balloon lowers the risk of cardiac tamponade. Balloon-based catheter ablation systems incorporating various energy sources are now clinically available, and developments are underway to achieve greater efficacy, efficiency, and safety. In addition, because extrapulmonary venous triggers can play a role in persistent AF, from the posterior wall of the LA, for example, existing ablation balloon-based approaches have advanced for effective treatment at these sites. The following is an overview of balloon catheter ablation systems and their use for treatment of AF, with a focus on currently available balloon technologies.

## 2. Cryoballoon Systems

### 2.1 Background

**Fig. 1.**
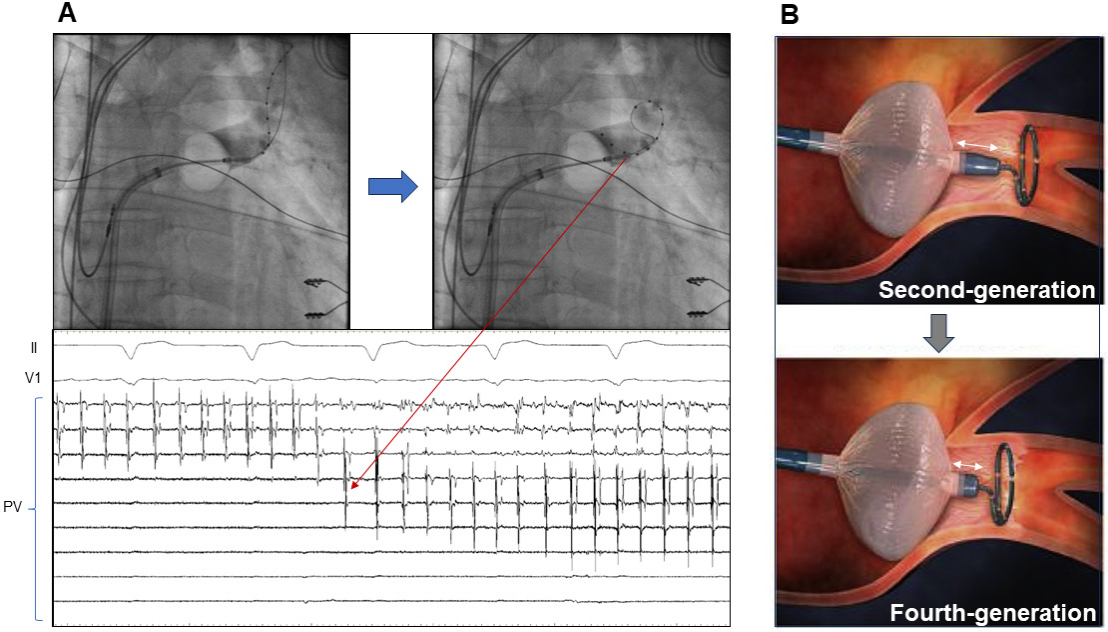
**Arctic Front cryoballoon (AFCB) system**. (A) The improvement in the pulmonary vein (PV) potential recordings by moving the catheter proximally immediately after the start of freezing. The short tip of the fourth-generation AFCB catheter contributes to the improved PV potential recording. (B) The white double-ended arrows show the tip length of the second- and fourth-generation AFCB catheters (13.5 mm vs. 8 mm). Adopted from Medtronic Japan.

Introduced over a decade ago, the Arctic Front cryoballoon (AFCB) system (Medtronic, Minneapolis, MN, USA) is the most often used and the most well-studied family of balloon devices for PVI. The AFCB system is composed of an over-the-wire catheter with an inflatable double-layer balloon at its distal end and a specialized 15F steerable sheath (FlexCath Advance; Medtronic). The lumen of the catheter is used to inject contrast and to deploy a dedicated spiral mapping catheter (Achieve; Medtronic) for recording PV electrograms. Liquid nitrous oxide is delivered into the system to induce hypothermia at the catheter/tissue interface, leading to cell injury and eventual cell necrosis [8], resulting in the formation of a nonconductive myocardial lesion. Cryoballoon (CB)-based ablation has undergone a technological evolution since 2003. The first-generation AFCB system featured four refrigerant jets positioned 7 mm from the balloon tip, with cooling occurring mainly along the balloon’s equator. The second-generation system (Arctic Front Advance; Medtronic) was redesigned to have eight refrigerant jets positioned 2.5 mm from the balloon tip, allowing for a more homogeneous cooling of the entire distal hemisphere of the balloon. The relatively long distal tip of the balloon catheter of the first-generation and second-generation systems (13.5 mm in the second-generation system) limited the proximal position of the circular mapping catheter and thus limited the real-time recording of PV potentials during the PVI. The third-generation system (Arctic Front Advance ST, Medtronic) with a 40% shorter tip of the catheter was introduced to address this drawback. Although the design of this balloon catheter significantly improved the PV recording rates, the differences in the temperature recording and reduced catheter stability led to the withdrawal of that system from the market shortly after its introduction (Fig. 1A) [9]. The fourth-generation AFCB system (Artic Front Advance Pro; Medtronic) with a reduced catheter tip length of 8 mm (Fig. 1B) was recently launched. Unlike those of the third-generation system, the positions of the injection coil and thermocouple were not changed from those of the second-generation system [10, 11]. The AFCB is available in two sizes (diameters): 23 mm and 28 mm.

Boston Scientific’s POLARx cryoablation balloon catheter has recently become commercially available. Although the overall design of the cryoballoon appears similar to the Medtronic Arctic Front system, the POLARx system has some significant differences that may alter the dose and ablation technique [12]. These features will be discussed later in this review.

### 2.2 CB-Based-PVI

PVI performed with the AFCB system proceeds as follows: The balloon is inflated outside the PV, then advanced to the ostium to occlude the PV; blood flow around the balloon can, by convective heat transfer, considerably limit the generation of cryolesions and prevent the creation of contiguous lesions. Therefore, it is necessary to selectively inject contrast medium through the distal port of the balloon catheter to occlude the targeted PV completely. The multipolar circular and spiral mapping catheter is placed either distal to the PV as a guidewire to assist in positioning the AFCB or proximal to the balloon tip for real-time recording of the PV electrogram during ablation. The latter is preferred because it allows recording the time from the start of cryoenergy delivery to the PVI (“time to isolation” [TTI]) and helps the operator adjust the time and frequency of freezing for maximum safety and efficacy. Once the spiral mapping catheter is in place, liquid nitrous oxide is supplied to the system, lowering the internal temperature of the balloon to –80 °C and producing a thermal lesion at the tissue contact site. Once cryoablation is started, the balloon adheres to the surrounding tissue during freezing, facilitating the catheter stability. This is particularly useful when ablation is performed at sites of potential catheter contact instability, such as the ridge region between the left superior PVs and left appendage. Tomographic imaging prior to the procedure may be useful.

The first-generation AFCB system yielded acute PVI rates of 92–100% [13]. However, the second-generation AFCB system improved the freezing characteristics, significantly improving the procedure’s effectiveness, with a higher incidence of a single-shot PVI, shorter TTI, and reduced procedure and fluoroscopy times [14, 15]. Among the patients who underwent an invasive mapping procedure 3 months after the index procedure performed with a second-generation AFCB system, 91% of the PVs remained electrically isolated, and all PVs were isolated in 79% of patients [16]. The TTI-based dosing of the cryoenergy delivery also resulted in reduced cooling and procedure times compared to the old protocol, with similar treatment effects [17, 18]. The fourth-generation system is expected to further increase the frequency of the TTI recording and thereby improve the ablation procedure [19]. The latest fourth-generation AFCB ablation and TTI recordings are shown in Fig. 1. Ablation of the right lower PV is often the greatest challenge of the CB ablation procedure, especially in cases in which the PV has a low balloon release position relative to the transseptal puncture site and the position of the balloon relative to the vein is not optimal. In addition, the right inferior PV is often relatively small in diameter, resulting in a large portion of the balloon surface being enveloped by flowing blood, which can hinder optimal cooling. This would explain why the right lower PV is the most frequent site of electrical reconnections after an AFCB system-based PVI [16, 19, 20].

Patient-related factors that make the PVI with an AFCB difficult include a significant enlargement of the PV ostium [21, 22], the degree of ovality [21], a ridge shape [23], and the PV bifurcation angle [24, 25]. It has been suggested that these conditions may lead to a mismatch between the PV ostium and balloon size, which may exacerbate the occlusion conditions, preventing the TTI in the short term and causing PV-LA reconnections in the remote phase [26]. The new POLARx cryoablation balloon catheter, with its softer balloon material and improved balloon compliance during freezing, has the potential to overcome these factors.

### 2.3 Clinical Outcomes

The randomized multicenter FIRE AND ICE trial stands as the most prominent trial to date comparing two ablation strategies for paroxysmal AF treatment [7]. This trial demonstrated the noninferiority of an AFCB vs. RF-based PVI with respect to the safety and efficacy. The smaller randomized multicenter FREEZE AF and CIRCA-DOSE (cryoballoon vs. irrigated radiofrequency catheter ablation) trials led to the same conclusion, with FREEZE AF demonstrating the noninferiority of a cryoablation-based PVI to a nRF-based PVI at 30 months [27, 28], and this finding held up even after a long-term follow-up of 30 months [29]. As a result, recent consensus documents have been updated to recommend using either RF or AFCB ablation as the catheter ablation method for the PVI [30, 31].

The STOP Persistent AF, an international multicenter, non-randomized, single-arm, open-label trial, evaluated the benefit of AFCB ablation for persistent AF [32]. The primary efficacy and primary safety endpoints were 54.8% and 0.61%, respectively, meeting the set targets. Those results led to the recent approval of insurance coverage of AFCB-based ablation for persistent AF in Japan. In addition, three randomized trials, the STOP AF First [33], EARLY-AF [34], and Cryo-FIRST [35], comparing cryoablation as a first-line therapy against antiarrhythmic drug pharmacotherapy, have recently been reported. The EARLY AF and Cryo-FIRST trials showed a significantly improved quality of life with AFCB-based ablation, establishing AFCB ablation as a first-line therapy that should precede drug therapy. Thus, the AFCB system is a balloon device unrivaled in the wealth of evidence supporting its use.

### 2.4 The New POLARx Cryoablation System 

The newly launched POLARx cryoablation system (Boston Scientific, St Paul, MN, USA) has a novel design and incorporates state-of-the-art technical features [36]. One feature of the POLARx system is that it maintains a constant balloon pressure throughout the inflation and freezing cycles, which can reduce the rate at which the balloon disengages from the PV antrum during the freezing energy delivery (pop-out phenomenon) [36, 37]. In addition, the POLARx system aims to increase the operator comfort during the procedure by providing instant control of the inflation and deflation, energy delivery, and double-stop maneuvers via a foot pedal and remote-control unit, as well as TTI recording. The POLARx system has a similar efficacy in achieving the vein occlusion and isolation and safety profile when compared to the AFCB system. The procedure time, fluoroscopy time, and cumulative freeze duration are significantly lower with the POLARx system [38]. In addition, the POLARx CB had a higher rate of real-time electrical recording of the PV potentials and a significantly lower balloon temperature as compared to the AFCB [36]. In the latest prospective studies, the new POLARx CB has shown a similar safety, efficacy and 1-year recurrence-free survival as compared to the AF-CB4 [39, 40].

## 3. Hot Balloon Systems

### 3.1 Background

The development of the RF hot balloon (HB) systems began in 2000 [41, 42]. After the clinical trials conducted by the PMDA (Pharmaceuticals and Medical Devices Agency), including a 17-center prospective, randomized, multicenter pivotal study [43], the HotBalloon ablation catheter (Toray Industries Inc., Tokyo, Japan) was granted manufacturing and marketing approval in Japan in November 2015.

**Fig. 2.**
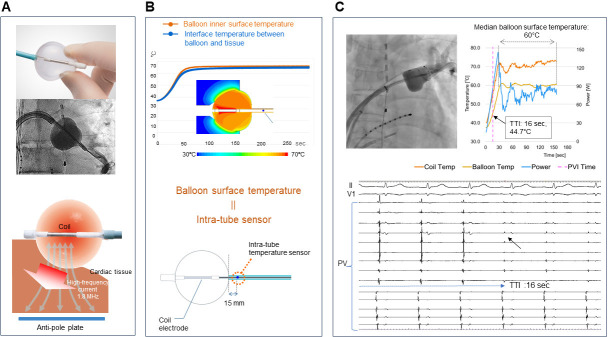
**Hot balloon system**. (A) The balloon compliance is maintained during ablation. The temperature of the liquid inside the balloon rises as radiofrequency waves flow from the coil inside the balloon to the anti-pole plate. Conductive heating has a thermal effect on the myocardium. (B) [Upper panel] The results of the computer-aided thermo-fluid analysis. [Lower panel] An additional temperature sensor lies 15 mm from the south pole of the balloon on the catheter shaft. The tissue temperature corresponds to the estimated balloon surface temperature recorded from the temperature probe (upper panel). (C) Fluoroscopic image and intracardiac potentials, internal balloon and surface temperature dynamics during the electrical isolation of the left superior PV, with a TTI of 16 sec and a median balloon surface temperature of 60 °C. PV, pulmonary vein; TTI, time to isolation; PVI, pulmonary vein isolation. Adopted from Toray Inc.

The HB system features an elastic balloon. In the center of the balloon is a shaft-mounted coil electrode for delivery of RF energy, and a temperature sensor is also mounted on the shaft (Fig. 2A). When the physician injects 10–20 mL of solution, the balloon is inflated to 26–33 mm (it can be inflated to 35 mm if the balloon membrane is tightly adhered to the PV tissue). Most of the irradiated RF energy is used to heat the solution filling the balloon. By maintaining the temperature of the coil electrode at 70 °C, the surface temperature of the entire balloon is 62–65 °C, and the balloon heats up, whereas the tissue temperature remains below 60 °C (Fig. 2A), thus minimizing damage to adjacent organs. This is in contrast to what occurs when catheters are used for conventional point-by-point RF ablation, where deep intramyocardial tissue temperatures are difficult to predict due to the cooling effects of blood flow on conductive heating that is responsible for large proportion of the ultimate lesion size. An extracorporeal agitation pump constantly agitates the fluid in the HB to maintain a uniform surface temperature. The system creates a circumferentially uniform lesion in the PV. The balloon is elastic and fits snugly regardless of the PV geometry, thus minimizing the anatomical constraints encountered under different ablation strategies.

The solution filling the balloon is an ionic liquid (a mixture of saline and contrast medium). As RF energy passes between the coil electrode and the return electrode pad on the patient’s back, the RF current is concentrated around the coil electrode, generating Joule heating, which raises the temperature of the solution in the balloon. Meanwhile, the extracorporeal agitation pump constantly agitates the solution inside the balloon, with the energy transfer keeping the surface temperature of the balloon uniform.

Target temperatures have been carefully determined on the basis of the results of several animal-based and phantom-based tests plus clinical experience in investigator-led trials. We further validated the tissue temperatures in animal models, and we found a significant inverse correlation between the recorded tissue temperatures and distance from the balloon surface (r = –0.67; *p* < 0.001) [44]. The tissue temperatures did not exceed the balloon set temperatures; the best distance cut-off value for achieving a lethal tissue temperature (above 50 °C) was 3.6 mm [44]. In conclusion, the data on the HB set temperatures, energy delivery times, and tissue temperatures support the clinical efficacy and safety of use of the current HB techniques in patients with AF.

### 3.2 HB-Based PVI

Different ablation protocols have been proposed for the HB-based PVI. The first multicenter randomized trial comparing the HB-based PVI with antiarrhythmic drug therapy followed a three-step protocol [43]. The balloon was first placed in full contact with the PV ostium to occlude the targeted PV; after the PV ostial ablation, the balloon was slightly inflated to contact a more antral region, and the ablation was performed repeatedly; the balloon was then positioned on the carina [43]. Among 100 patients randomized to interventional treatment, HB ablation resulted in an acute isolation of 98.0% of the PVs (392 of 400) and in 93% of the patients. Although the rate of an acute PVI achieved by this technique was promising, PV stenosis was observed in 5.2% of the patients with a decrease in the PV diameter of >70% on 3D-computed tomography (CT) after ablation. In all 100 cases, less than 8 mL of fluid was injected into the balloon, resulting in smaller balloon diameters and, consequently, a tendency for the balloon to be located more deeply in the PV.

A single-shot ablation protocol has recently been proposed to avoid applying energy deep within the PV [45]. For that purpose, the balloon is inflated to the maximum size that achieves an occlusion of the targeted PV as assessed by selective angiography. The initial injection volume tested was 10 mL. Particular care was paid to applying pressure in the coaxial direction to ensure that the HB did not dislodge from the PV antral region. Ablation was performed at 70 °C for 3.5 min in the right upper PV, 70 °C for 3 min in the right lower PV, 70 °C for 2.0–2.5 min in the left lower PV, and 70 °C for 4 min in the left upper PV. When this method was used in 61 paroxysmal AF patients, an acute PVI was achieved for 200 of 241 PVs (83%) in 31 patients (51%). Touch-up ablation with an irrigated RF ablation catheter was performed if the PVs could not be isolated after a maximum of two applications. Those “touch-ups” were required for 41%, 5%, 16%, and 10% of the left upper PVs, left lower PVs, right upper PVs, and right inferior PVs, respectively. Most PVs requiring additional touch-up ablation had a single gap (89%). Five percent of the patients who underwent a post-procedure CT assessment were found to have severe (>70%) PV stenosis. Suruga *et al*. [46] compared CB and HB. The need for additional touch-up ablation was more frequent in the HBA group than in the CBA group and, as reported above, touch-up was more frequent in the left upper PVs [46]. The frequency of touch-ups was high with the first generation HBs, leaving issues to be addressed to increase the single-shot success rate.

Second-generation HBs are now able to provide an estimate of the balloon surface temperature (BST) (Fig. 2B,C) [47]. The PVI can now be performed by using a combination of the TTI and BST indexed by the PV potentials recorded from a circular catheter placed in the LA via a second sheath. We have performed the HB-based PVI by this method [47]. Acute isolation was achieved for 88% (106/120) of PVs with a single HB shot. The real-time BST and PV potentials were recorded in all cases; the mean BST at the time of the PVI was 49.4 °C, and an acute re-conduction was observed in most cases (86%, 12/14) in which the single-shot technique was not effective; the TTI (23.1 ± 8.7 sec vs. 36.3 ± 9. 3 sec, *p* < 0.01) and median BST (59.9 ± 2.6 °C vs. 55.7 ± 1.9 °C, *p* < 0.01) differed significantly between the cases in which a PVI was achieved and those in which it was not. The optimal median cut-off BST for achieving a PVI with a single injection was >58.7 °C (sensitivity 67.0%, specificity 100%). These data suggest that real-time BST monitoring during the energy application helps predict the achievement of an acute PVI by a single shot during HB ablation.

### 3.3 Clinical Outcomes

In a prospective, randomized, PMDA clinical trial involving 17 centers in Japan, the prevalence of normal sinus rhythm at 12 months was 59% among the patients undergoing a HotBalloon-based ablation. The resulting efficacy was significantly superior to that of drug treatment in the control group [43]. Following this clinical trial, the device was approved in Japan in 2015 to treat drug-resistant paroxysmal AF.

Several years after the clinical trial, a post-marketing study evaluated the efficacy and safety of HB treatment for paroxysmal AF in a real-world clinical setting [48]. It was a single-arm, multicenter observational study with a post-ablation observation period of 48 weeks. Forty-six centers in Japan were involved, and the achievement of a PVI and AF non-recurrence rates were assessed. Adverse events were also observed. AF events were defined as recurrence or re-ablation of AF from 12 to 48 weeks after the HB ablation. The final PVI success rate was 99.0% (486/491), and achievement of a PVI by balloon treatment alone was 77.3% (1499/1938 of all PVs). The cumulative AF-free recurrence rates were 94.1% at 24 weeks and 87.8% at 48 weeks. Ablation-related adverse events occurred in 2.6% (14/530) of patients, the most common being pericardial effusion (0.8%, 4/530).

Real-world data reflecting the efficacy and safety of the HB-based PVI in clinical practice have recently been reported [49]. A multicenter prospective registry study included patients undergoing an HB-PVI of AF, with the HBs being size-adjustable to treat different types of AF. The primary endpoint was the AF-free survival 12 months after the PVI. Of the 679 patients enrolled, 613 (90.3% [370 with paroxysmal AF, 136 with persistent AF, and 107 with long-term AF]) underwent an initial HB-PVI; an acute PVI was achieved by the HB alone in 55.6% of patients and for 83.5% of the PVs. The acute PVI rate was highest among the patients with paroxysmal AF and that were treated at centers with more cumulative experience; antiarrhythmic drugs were prescribed for 47.5% of the patients after 3 months; the recurrence-free survival at 12 months was 83.7%. Although antiarrhythmic drugs were used in about half of the patients and the arrhythmia-free rate at 12 months was acceptable, further improvement is needed before we can say an acute PVI is satisfactorily achieved by HB-based ablation. A recent report on the lesion durability showed that 83.5% of the PVs had a durable PVI, with a particularly high tendency in the lower PVs [50]. The HB system is only used in Japan and one of the current problems is the need for systematic and robust comparisons with other systems, which is an issue for the future.

## 4. Laser Balloon Systems

### 4.1 Background

**Fig. 3.**
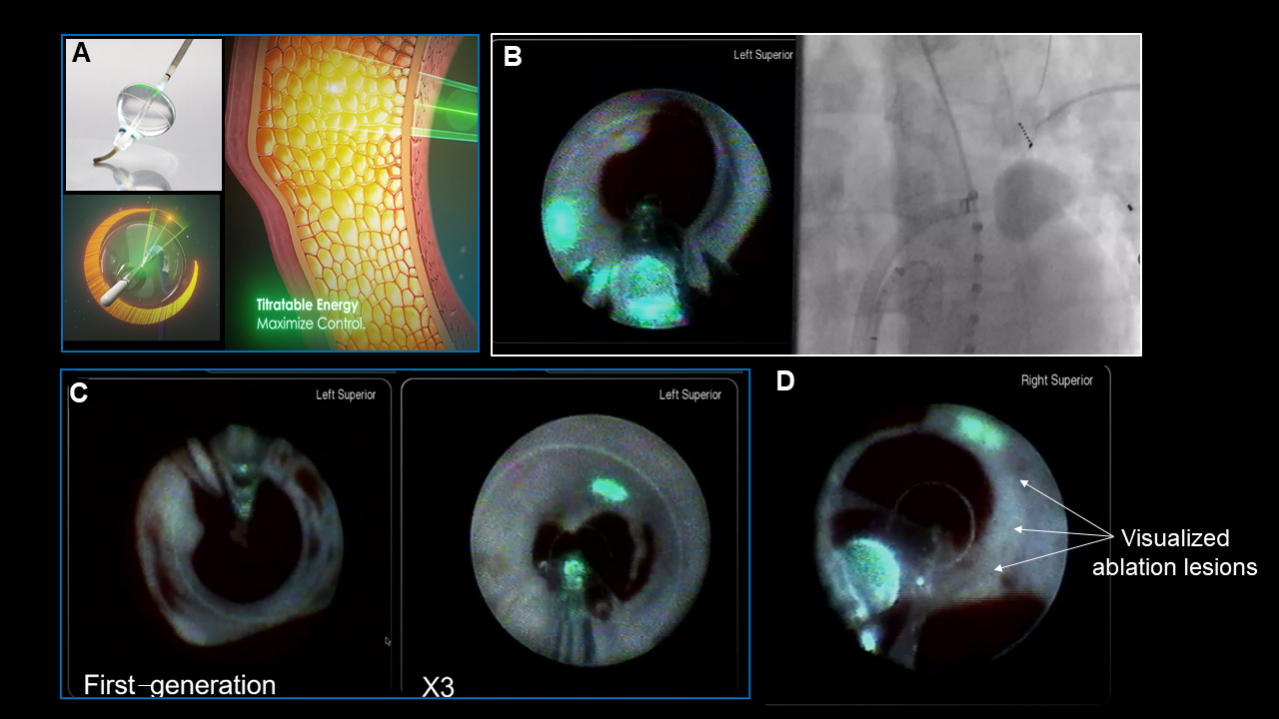
**Laser balloon system**. (A) The third-generation HeartLight X3 system is equipped with a motorized, fully automated circumferential energy delivery system. Laser energy penetrates the endocardium and produces a thermal effect from the mid-myocardium. (B) Fluoroscopic and endoscopic images of a balloon positioned within the left superior pulmonary vein (PV). (C) Endoscopic images obtained with the use of first- and third-generation laser balloon systems. (D) Direct endoscopic visualization of a laser ablation lesion. Adopted form Japan Lifeline.

The visually guided laser balloon (LB) system (HeartLight; CardioFocus Inc., Marlborough, MA, USA) is a clinically available balloon device equipped with a 2F endoscope that allows direct visualization of the PVs and uses 980 nm laser light for the creation of thermal lesions (Fig. 3A,B). The balloon is filled with deuterium oxide ($D_{2}$O), and laser energy is delivered from the balloon, which is continuously flushed and compliant. The laser light is not absorbed by the $D_{2}$O, resulting in volumetric heating of the target tissue and ultimate cell necrosis. Direct energy application to the blood pool should be avoided because thrombus formation can result. The compliant balloons can be inflated to various diameters ranging from 9 to 35 mm to overcome this obstacle. This provides optimal sealing of the target PV and a bloodless interface for ablation. A 2F fiberoptic endoscope is a unique feature that allows for real-time visualization of the PV ostial region and monitoring of peri-balloon contact to guide the AF treatment. The laser covers an arc of 30°, so the lesions are deployed point-by-point; application overlap is necessary to create a continuous lesion around the PV ostium. The tracking software visualizes the preceding ablation sites and facilitates a circumferential PVI. The energy setting is adjustable at 5.5–15 W and 20–30 sec output, and can thus be adapted to the thickness of the underlying tissue and proximity of the esophagus and respiratory nerves. Laser projection can be controlled independent of the balloon position, allowing the circumferential line to be customized around the PV ostium. The latest system (HeartLight X3 system, CardioFocus) includes a motor control unit capable of continuously moving the laser arc in a circumferentially at a speed of 2°/sec in a “drag and burn” fashion to accelerate the PVI [51]. It is important to note that only two laser energy output settings can be selected for this function: 13W or 15W. In addition, the balloon compliance has been improved, blood can be more easily excluded, and the PVI can be performed with a clear field of view (Fig. 3C,D). Furthermore, direct endoscopic visualization of the lesion is now possible. Compared to CBs, LBs are pear-shaped rather than spherical and are variable in size and compliance. Thus, it has the potential to adapt to a wider range of PV morphologies. A recent prospective randomized trial found that procedure times were significantly shorter with the CB than with the LB, but there was no difference in the fluoroscopy radiation dose [52]. The latest X3 system has the potential to reduce the procedure time.

### 4.2 LB-Based PVI

After a transseptal LA access is obtained, the LB is advanced into the LA through a 15F steerable sheath (CardioFocus). Pre-CT imaging or intraoperative PV angiography is recommended to assess the individual anatomical structures. During the PVI, the operator inflates the LB and adjusts the balloon diameter for an optimal PV occlusion. Each PV should be isolated individually rather than forming a wide circumferential lesion around the ipsilateral PV antrum [53]. Laser irradiation is performed with a 30–50% overlap around the PVs under direct guidance with visual observation; using a high energy setting of 8.5 W increases the efficacy of the LB-based PVI without the loss of the safety and reduces the procedure time [54, 55]. If the PV occlusion is not optimal, the laser energy may need to be reduced for any applications near a blood pool. The latest X3 system mentioned above has a motor that allows automatic circumnavigation of the catheter route in approximately 160 sec, which is expected to further reduce the procedure time [51].

The LB has no recording electrodes and no specialized mapping catheter. Therefore, a separate ring catheter is used to confirm a successful PVI. According to previously reported studies, the LB-based PVI takes 60–120 min, and 97–100% of the PVs are acutely isolated [56, 57]. The lesion durability was good, and 86% of the PVs remained electrically isolated in patients who underwent another interventional mapping procedure 3 months after the index procedure [58]. Another study demonstrated a ring catheter concurrently mounted on an LB to determine how much ablation coverage was required to achieve the PVI (i.e., whether all PVs required 360° full circumferential lesion formation). Surprisingly, after an upper full-perimeter ablation, the PVI was achieved in more than half of the lower PVs with a laser lesioning of less than half the circumference. This suggests that the crosstalk phenomenon resulting from delivery of laser energy to the carina region during an upper PVI may have been involved, supporting the notion of deep penetration of laser energy into the tissue [59].

### 4.3 Clinical Outcomes

In the second-generation system, the LB ablation yielded a clinical outcome comparable to that of an AFCB-based PVI [60]. In a strict follow-up with an implantable cardiac monitor (ICM), LB ablation alone exhibited a 66.9% freedom from any atrial arrhythmia [61] that was comparable to CB ablation, and when using propensity score matching, it had comparable outcomes [62]. The latest X3 system and first-generation (HeartLight system) were compared in a European two-center study. The RAPID mode with a motor-driven mechanism could be used for almost all PVs and produced continuous lesions quickly. The AF free rate at 1 year was 61.7% for RF ablation, 61.1% for HeartLight LB ablation, and 71.9% for X3-based ablation. The use of the X3 vs. HeartLight resulted in a significant reduction in the procedure time while maintaining the efficacy and safety [51, 63]. In a multicenter randomized controlled trial comparing RF ablation with HeartLight-based ablation for persistent AF, the results of the two treatment strategies were comparable, with resolution of the AF in approximately 70% of patients at 1 year [64]. Data on the long-term outcomes after the PVI performed with X3 are not yet available. However, in the latest report, the one-year results were good at 93.7% in patients with paroxysmal AF and 81.3% in those with persistent AF, with a reduced procedure time and high first-pass isolation rate (91.1%) [65].

## 5. Features of the Three Balloon Systems and Potential Clinical Complications Associated with Their Use

### 5.1 Features of Each of the Three Balloon Systems. 

**Fig. 4.**
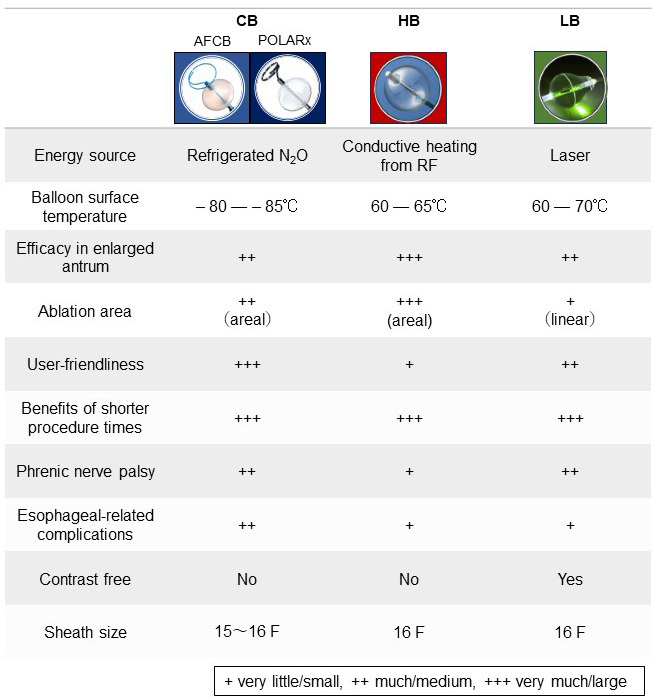
**Features of the three currently available balloon ablation systems**. CB, cryoballoon; HB, hot balloon; LB, laser balloon; AFCB, Arctic Front cryoballoon; RF, radiofrequency. Adopted from Medtronic Japan, Toray Inc, and Japan Lifeline.

The features of each of the three balloon systems described above are shown side-by-side in Fig. 4. The potential clinical complications are described below.

### 5.2 Phrenic Nerve Palsy

As the cooling properties of second-generation AFCBs have improved, an increase in right-sided phrenic nerve injury causing diaphragmatic hemiparesis has been observed, the incidence of which was approximately 3% in a recent clinical trial [7, 66, 67]. This risk increases with an increasing pressure on the balloon during cryofreezing but can be significantly reduced by changing to a more antral approach or by using a large size (28 mm) balloon [68]. Pacing maneuvers from the right diaphragmatic nerve via the subclavian vein or superior vena cava and close monitoring of the diaphragmatic stretch can help operators detect diaphragmatic nerve dysfunction promptly. Furthermore, a decreased diaphragm compound motor potential amplitude predicts paralysis during respiratory nerve pacing, requiring immediate discontinuation of the cryoenergy delivery [69]. The amplitude of the compound motor potential can be measured by repositioning two surface electrodes across the diaphragm. Most cases of diaphragmatic nerve palsy resolve within 12 months after ablation.

For HB ablation, as for CB ablation, phrenic nerve pacing is routinely performed from the superior vena cava during the ablation of the right PVs. Phrenic nerve palsy tends to occur less frequently after HB ablation than after CB ablation, with an incidence of 3.7% reported at the time of the clinical trials [43], 0.9% reported in the post-marketing surveillance [48], and 1.0% reported most recently [49].

With the first-generation LB system, the reported incidence of diaphragmatic paralysis was significantly higher among LB-treated patients than among RF-treated patients (3.5% vs. 0.6%), despite diaphragmatic pacing during the right superior PV ablation. Recent reports show a decrease to around 1.5%. Although this incidence appears lower than with CB systems, phrenic nerve palsy after LB ablation has a low recovery rate during the follow-up [70, 71]. Thus, continuous monitoring of the phrenic nerve function is essential, especially when a right-sided PV is approached, and a large LB diameter is desirable to avoid unintentional ablation deep within the PV.

### 5.3 Esophageal Injury

CB-induced esophageal injury is rare, and an association between an LIPV intervention and a prolonged ablation time has been observed. John *et al*. [72] found 11 cases among 120,000 patients from the Manufacturer and User Facility Device Experience database, publications, and manufacturers’ databases, i.e., fewer than 0.1% of cases. The balloon dilation time was significantly longer in the patients with fistulae (238.8 ± 54.8 sec compared with 178.1 ± 37.5 sec in the non-esophageal fistula group, *p* < 0.001). Although not proven in this study, a balloon nadir temperature above 60 °C may also increase the risk of injury and should be avoided. Monitoring the esophageal lumen temperature during ablation is controversial but may help alert the operator to potential thermal damage to the esophagus.

The HB system is the only balloon device that incorporates active cooling of the esophagus, effectively lowering the risk of thermal damage to the esophagus caused by conductive heating [43, 44, 73]. In the past, a cooled saline solution was injected manually, but in recent years an automatic infusion and return system has been adopted. Although no left atrial-esophageal fistulae have resulted from clinical trials or during post-marketing surveillance, a recent registry trial identified a single case (0.2%) of a left atrial-esophageal fistula. It is unclear whether the esophageal cooling system was properly operated in that case.

During LB ablation, it is recommended that an oral probe be used to monitor the esophageal temperature. According to data from a small case series, the risk of thermal injury was low if the esophageal temperature did not exceed 39 °C. Higher esophageal temperatures may result in more severe mucosal injury, as is known for other sources of energy [74]. However, the pooled data have shown no cases of atrio-esophageal fistulae after LB ablation [75].

### 5.4 PV Stenosis

With CB-based ablation, the freeze times were relatively long in the early years, and remote-phase symptomatic PV stenosis was observed in sporadic cases [76, 77]. Tokutake *et al*. [78] reported severe (>75% reduction) PV stenosis in the remote phase in 1.3% of PVs as measured on CT. Balloon angioplasty and stenting have been reported for symptomatic PV stenosis resulting from CB ablation and HB ablation [77, 79, 80], but there are currently no reports of balloon angioplasty for PV stenosis resulting from LB ablation. This is likely because the thermal effect of CB ablation and HB ablation extends to the distal PV due to the nature of the respective systems [81]. According to a recent report by Tokuda *et al*. [82], the incidence of symptomatic PV stenosis across the three balloon systems was only 0.3%, and the incidence tended to decrease with the operators’ experience.

## 6. Conclusions

The balloon catheter ablation systems currently available and used particularly for PVI are outlined above. Each system is designed to function under one of two general principles. CB and HB systems hinge on a single, homogeneous areal heat transfer effect, allowing rapid energy delivery to the target tissue. Such systems, in comparison to others, may produce lesions of greater uniformity. However, the energy cannot be modulated at specific regions to prevent “undertreatment” of relatively thick atrial muscle or “overtreatment” of relatively thin atrial muscle. LB systems address this drawback by tailoring the energy setting to different anatomical regions. In addition, pulsed field ablation (PFA) has recently emerged as a promising non-thermal ablation modality for the treatment of AF. Several balloon-based PFA systems are under development that will attempt to use improved contact for better lesion delivery.

In conclusion, AF ablation with balloon technologies is undergoing considerable growth, diversity, and modernization with the development of new products and the maturation of well-established techniques. Additional clinical data and accumulated experience on the latest balloon systems are needed before final conclusions can be drawn on the precise role of balloon catheters in AF ablation.
